# Effects of bamboo leaf extract on the production performance, rumen fermentation parameters, and rumen bacterial communities of heat-stressed dairy cows

**DOI:** 10.5713/ab.20.0527

**Published:** 2021-01-14

**Authors:** Yi Li, Luoyun Fang, Fuguang Xue, Shengyong Mao, Benhai Xiong, Zhu Ma, Linshu Jiang

**Affiliations:** 1 Beijing Key Laboratory for Dairy Cow Nutrition, Beijing University of Agriculture, Beijing 102206, China; 2 Jiangxi Province Key Laboratory of Animal Nutrition/Engineering Research Center of Feed Development, Jiangxi Agricultural University, Nanchang, Jiangxi 330045, China; 3 Jiangsu Key Laboratory of Gastrointestinal Nutrition and Animal Health, Laboratory of Gastrointestinal Microbiology, College of Animal Science and Technology, Nanjing Agricultural University, Nanjing 210095, China; 4 State Key Laboratory of Animal Nutrition, Institute of Animal Science, Chinese Academy of Agricultural Sciences, Beijing 100193, China; 5 Beijing Dairy Cattle Center, Beijing 100085, China

**Keywords:** Bamboo Leaf Extract, Dairy Cows, Heat Stress, Production Performance, Rumen Bacterial Communities, Rumen Fermentation

## Abstract

**Objective:**

An experiment was conducted to evaluate the effects of bamboo leaf extract (BLE) on the production performance, rumen fermentation parameters, and rumen bacterial communities of heat-stressed dairy cows.

**Methods:**

The experiment comprised a 14-day adaptation period and a 21-day experimental period and was conducted in a high-temperature and humidity environment (daily mean ambient temperature = 33.5°C±1.3°C; daily mean relative humidity = 64.9%±0.8%, daily mean temperature-humidity index = 86.2±0.4). Twelve Holstein dairy cows were randomly allocated into two groups. A total mixed ration supplemented with BLE at 0 (CON) and 1.3 g/kg dry matter (DM) were fed, respectively. Feed intake and milk yield were recorded daily. Milk samples were collected on 1, 11, and 21 d of the experimental period to analyze milk performance. Rumen fluid samples were collected on 21 d of the experimental period to analyze rumen fermentation parameters and rumen bacterial communities.

**Results:**

Compared with the control group, supplementation of BLE increased milk yield (p<0.01), milk fat yield (p = 0.04), 4% fat-corrected milk (p<0.01) and milk fat content (p<0.01); reduced somatic cell count (p<0.01). No differences in DM intake and milk protein or lactose content were observed between two groups. Supplementation of BLE also increased the rumen total volatile fatty acid (p<0.01), acetate (p<0.01), butyrate (p<0.01), and valerate (p = 0.05) concentrations. However, no significant effects were observed on rumen pH, ammonia nitrogen, propionate, acetate/propionate ratio, isobutyrate, or isovalerate. Furthermore, BLE increased the rumen bacterial abundance and the diversity of the rumen bacterial community. The BLE reduced the *Firmicutes*/*Bacteroidetes* abundance ratio and increased the abundances of Butyrivibrio_2 (p<0.01) and Ruminococcus_2 (p<0.01).

**Conclusion:**

The BLE supplementation at 1.3 g/kg DM could improve production performance and rumen fermentation in dairy cows during heat stress.

## INTRODUCTION

Heat stress causes great economic loss to the livestock industry worldwide, particularly the dairy industry [1]. Previous studies have shown that heat stress inhibits the peristaltic contractions of the rumen, reduces volatile fatty acid (VFA) concentrations, increases the abundance of harmful bacteria, and reduces the production performance of cows [2]. Therefore, it is important to identify effective means of relieving heat stress in cows.

Bamboo leaf is a traditional folk medicine and usually used to treat heat stroke, fevers and enteritis in East Asian countries including China, Japan and Korea. Bamboo leaf flavonoids have been reported as the major bioactive substances and are used as the chemical marker for the quality evaluation of bamboo leaf extract (BLE) [3]. The BLE extract has been shown to improve the carcass performance of broilers, and the meat quality of heat-stressed broilers, and to reduce the incidence of diarrhea in weaned pigs [4,5]. Additionally, our preliminary study found that supplementation of BLE at 1.3 g/kg dry matter (DM) was the optimal level, which improved the lactation performance, antioxidant capacity, and immunity of non-heat stressed cows [6]. However, there have been no reports of the effects of BLE in heat-stressed cows.

Previous studies have shown that the rumen bacterial communities influence the production performance of dairy cows by affecting feed digestibility and energy supply [7]. Flavonoids may regulate the rumen flora. Flavone, myricetin, catechin, rutin and kaempferol increase the population of rumen microbes, while naringin and quercetin reduce the total populations of protozoa and methanogens *in vitro* [8]. Additionally, the ciliate, *Ruminobacter albus* and *R. flavefaciens* populations decreased and the *Fibrobacter succinogenes* diversity increased in response to flavonoid-rich plant extracts [9]. Therefore, we hypothesized that BLE may improve rumen fermentation in heat-stressed cows by regulating the structure of the rumen bacterial flora, which would improve the production performance of the cows. In the present study, we aimed to determine the effects of BLE supplementation on the production performance, rumen fermentation parameters, and rumen bacterial structure of heat-stressed dairy cows.

## MATERIALS AND METHODS

### Animal care

All the experimental protocols performed in this study were approved by the Animal Ethics Committee of the Beijing University of Agriculture (No. BUA20180324).

### Animal management

The cows were housed in a ventilated free-stall barn. To ensure the total mixed ration (TMR) was fresh and available for the cows for at least 20 hours a day, they were fed three times daily at 07:00, 13:00, and 18:00 *ad libitum*. Automatic feeding equipment (Insentec Co., Amsterdam, The Netherlands) was used to fed each cow in a separate trough and recorded daily feed intake. Cows were milked three times daily at 09:00, 15:00, and 20:00. using an Afimilk facility (90° Side-by-Side Parallel Stall Construction, Afimilk Co., Tel Aviv-Yafo, Israel).

### Bamboo leaf extract

The BLE was produced by Senfu Biological Co., Ltd. (Xi’an, China). Bamboo leaf samples were extracted three times with 95% aqueous ethanol at room temperature (24 h×20 L). The filtrates were combined and concentrated under reduced pressure for removal of the organic solvent using rotary evaporator (RE-2000B, Ruide Analytical Instruments Co. Ltd., Henan, China). After the extract was completely dried, it was suspended in water and lyophilized using a freeze dryer (FD-1A-50, Shunzhi Analytical Instruments Co. Ltd., Beijing, China) to yield a brown residue, which was the BLE [10]. The BLE content was determined by rapid resolution liquid chromatography [11], and it contained 41.2% total flavonoids, 20.5% crude ash, 13.2% crude protein (CP), 15.4% water-soluble polysaccharide, and 9.7% other components.

### Experimental design and diets

The experiment comprised a 14-day adaptation period and a 21-day experimental period. We conducted the experiment at a dairy farm in Beijing between July and August, when the environmental temperature and humidity were relatively high (daily mean ambient temperature = 33.5°C± 1.3°C; daily mean relative humidity = 64.9%±0.8%, daily mean temperature-humidity index = 86.2±0.4). Twelve Holstein dairy cows were used in a completely randomized design experiment with two diets (n = 6). The cows in each group were matched according to their parity (3.3±0.3), body condition score (3.4±0.1), initial body mass (559.2±37.3 kg), milk yield (38.4±0.8 kg/d), and the number of days in milk (185.7 ±16.9 days). All of the cows received the same basal TMR, which was formulated according to NRC (2001) [12] to meet or exceed nutritional requirements. The ingredients and chemical composition of the base diet are shown in Table 1. The two diets were TMR supplemented with BLE at 0 (CON) and at 1.3 g/kg DM (BLE), respectively.

### Diet sampling and analysis

Samples of each diet were collected twice per week, stored at −20°C, and composited weekly for the analysis of DM, CP, ether extract (EE), neutral detergent fiber (NDF), acid detergent fiber (ADF), ash, calcium (Ca), and phosphorus (P). We calculated dry matter intake (DMI) according to the feed intake recorded by Automatic feeding equipment (Insentec Co., The Netherlands) and DM obtained through dietary analysis. The DM content was determined by oven drying at 105°C to constant mass (method 930.15, AOAC) [13], the CP content was determined using Kjeldahl nitrogen analysis (method 945.16, AOAC) [13], and the EE content was determined using a Soxhlet extractor (method 945.16, AOAC) [13]. NDF and ADF were analyzed using heat-stable amylase (A3306, Sigma Chemical Co., St. Louis, MO, USA) and sodium sulfite according to the procedure of Van Soest et al [14]. The ash content was measured by combustion using a muffle furnace (method 942.05, AOAC) [13]. A colorimetric method was used for the analysis of phosphorus (Spectrophotometer UV752N, Yoke Instrument Co. Ltd., Shanghai, China) and calcium was measured using atomic absorption spectrometry (PerkinElmer AAS800, Waltham, MA, USA).

### Measurement of ambient temperature and relative humidity

Ambient temperature (Td, °C) and relative humidity (RH, %) were measured using a temperature and humidity data-logger (ST-172; Fotronic Co., Melrose, MA, USA) at 15-min intervals over 24 h to calculate the temperature-humidity index (THI). The equation used to calculate THI was THI = 0.81Td+ (0.99Td–14.37) RH+46.3 [1]. Previous studies have shown that when THI is >72, dairy cows enter a state of heat stress [1]. In the present study, the daily mean THI was 86. Therefore, the cows were in a state of heat stress throughout the entire experimental period.

### Milk sampling and analysis

The Afimilk facility was used to record the milk yield of each cow. On the 1, 11, 21 d of the experimental period, we collected milk samples in 100-mL vials at each milking session, mixed them in proportion (morning:afternoon:evening = 4:3:3), and added 2-bromo-2-nitro-1,3-propylene glycol as a preservative. The milk samples were stored at 4°C before being sent to the Milk and Dairy Products Quality Supervision and Testing Center, Ministry of Agriculture (Beijing, China) for analysis of the milk protein, fat, lactose and somatic cell count (SCC) by mid-infrared spectroscopy (Fossomatic 4000, Foss Electric A/S, Hillerød, Denmark). The 4% fat-corrected milk (4% FCM) was calculated [15] as 4% FCM = M×(0.4+ 15F), where M is milk yield (kg) and F is milk fat ratio (%).

### Rumen fluid sampling and analysis

On the 21 d of the experimental period, a gastric rumen sampler was used to collect rumen fluid samples *via* the esophagus 2 h after the morning feed. The collected samples were strained through four layers of cheesecloth with a mesh size of 250 μm to obtain the rumen fluid. To reduce contamination with saliva, the first 250 mL of the rumen fluid was discarded. The pH of each rumen fluid sample was immediately measured using a portable pH meter (Testo 205, Testo AG, Lenzkirch, Germany). The rumen fluid was then divided into two parts, one of which was processed to analyze VFA and ammonia nitrogen (NH_3_-N) content, and the other was frozen in liquid nitrogen immediately after the addition of a stabilizer, and then stored at −80°C prior to DNA extraction. The individual and total VFA (TVFA) contents of the rumen fluid aliquots were determined by gas chromatography (GC-2010, Shimadzu, Kyoto, Japan), as described. The NH_3_-N content was determined by the indophenol method, as described.

### DNA preparation and sequencing

Bacterial DNA was extracted from samples using a Power Soil DNA Isolation Kit (MO BIO Laboratories, Carlsbad, CA, USA) with TRIzol agent, according to the manufacturer’s protocol. After reverse transcription, 16S rRNA primers were used to identify bacterial taxa (F: 5′-ACTCCTACGG GAGGCAGCA-3′; R: 5′-GGACTACHVGGGTWTCTAAT-3′. Polymerase chain reaction (PCR) amplification was performed in a total volume of 50 μL, which contained 10 μL buffer, 0.2 μL Q5 High-Fidelity DNA Polymerase, 10 μL High GC Enhancer, 1 μL dNTP, 10 μM of each primer, and 60 ng genomic DNA. The thermal cycling conditions were as follows: initial denaturation at 95°C for 5 min; then 25 cycles of 95°C for 1 min, 50°C for 1 min, and 72°C for 1 min; and a final extension at 72°C for 7 min. The PCR products from the first PCR were purified through VAHTSTM DNA Clean Beads. A seTNd round PCR was then performed in a 40 μL reaction volume that contained 20 μL 2×PHμsion HF master mix, 8 μL ddH2O, 10 μM each primer, and 10 μL PCR product from the first step. The thermal cycling conditions were an initial denaturation at 98°C for 30 s; followed by 10 cycles of 98°C for 10 s, 65°C for 30 s, min, and 72°C for 30 s; and a final extension at 72°C for 5 min. Finally, the PCR products were quantified using Quant-iT dsDNA HS Reagent and pooled.

High-throughput sequencing analysis of the bacterial rRNA genes was performed on the purified, pooled samples using the Illumina Hiseq 2500 platform (2×250 paired ends) at the Biomarker Technologies Corporation (Beijing, China). Quality filtering of the raw tags was performed under specific filtering conditions to obtain high-quality clean tags, according to the QIIME (V1.7.0) quality control process. The tags were then compared with a reference database using the UCHIME algorithm to detect chimeric sequences, which were removed. The remaining useful tags were sequenced using Uparse software (Uparse v7.0.1001). Sequences with >97% similarity were assigned to the same operational taxonomic units (OTUs), and then a representative sequence for each OTU was screened for further annotation. For each representative sequence, the GreenGene Database was used, with a ribosomal database project classifier algorithm, to annotate the taxonomic information. The abundances of the OTUs were normalized using a standard sequence number corresponding to the sample with the fewest sequences. Alpha diversity and beta diversity were then quantified using these normalized output data.

### Statistical analysis

The normal distribution of production performance, ruminal pH, VFA, and NH_3_-N was first confirmed using the SAS (version 9.2; SAS Institute Inc., Cary, NC, USA) procedure “proc univariate data=test normal”. Production performance was performed using the MIXED model in SAS. Ruminal pH, VFA and NH_3_-N were performed using Student’s t-test in SAS. The OTU abundances of rumen bacteria were first transformed to create normal distributed data using log2, and then Student’s t-test was used to analyze differences in the bacterial populations. Differences among treatments were considered to be significant at p<0.05, and as a tendency to be significant at 0.05≤p<0.10. Bar plots, principal coordinate analysis (PCoA), and hierarchical clustering analysis for the rumen bacterial populations were conducted using R package version 3.3.1. Spearman correlations between bacteria communities and DMI, milk yield and ruminal fermentation variables were calculated using the PROC CORR procedure of SAS. The relative abundances of all the bacterial phyla were used to conduct these analyses. A correlation matrix was created and visualized in a heat map format using R package version 3.3.1.

## RESULTS

### Production performance

As shown in Table 2, BLE supplementation at 1.3 g/kg DM was associated with higher milk yield (p<0.01), milk fat yield (p = 0.04), 4% FCM (p<0.01) and milk fat content (p<0.01); and lower SCC (p<0.01) than the control diet. However, no significant effect of BLE supplementation was found on DMI, milk protein or lactose content.

### Rumen fermentation parameters

As shown in Table 3, BLE supplementation at 1.3 g/kg DM was associated with higher TVFA (p<0.01), acetate (p<0.01), butyrate (p<0.01), and valerate (p = 0.05) concentrations than the control diet. However, no significant effect of BLE supplementation was found on rumen pH, the acetate/propionate ratio, the propionate, isobutyrate, isovalerate, or NH_3_-N concentrations.

### Sequencing information

**Figure 1:**
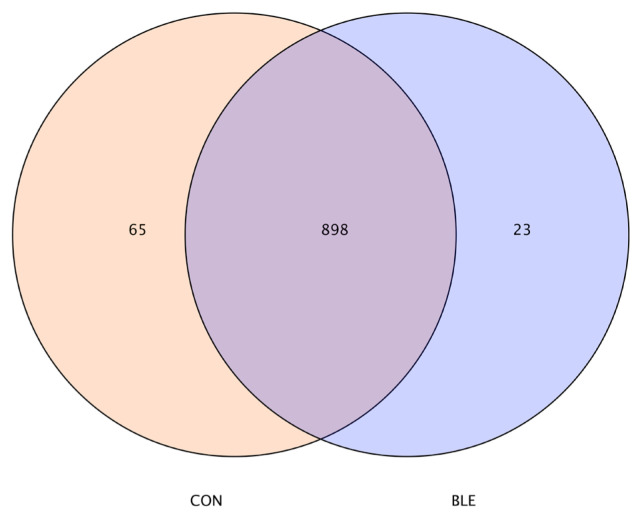
Distribution of operational taxonomic units (OTUs) among the two diets. CON, total mixed ration supplemented with bamboo leaf flavonoid (BLE) at 0 g/kg dry matter; BLE, total mixed ration supplemented with BLE at 1.3 g/kg dry matter. The overlapping part is the total number of OTUs among the two diets, while the non-overlapping part is the number of OTUs unique to each diet.

The bacterial 16S rRNA genes were amplified using the barcoded universal primers 338F and 806R, which span the V3–V4 hypervariable region. The number of sequencing reads per sample was approximately 62,009 and the mean length of each read was more than 420 bp. The fourteen phyla, and more than one hundred and sixty genera were identified. As shown in Figure 1, a total of 986 OUTs were produced by the two treatments, 963 OUTs were produced by the control group, and 921 OUTs were produced by the BLE group, among which 898 were in total.

### α-Diversity

All the identified bacteria were chosen for further analysis to investigate the effects of BLE on the rumen bacterial community. Alpha diversity was used to assess the complexity of the species diversity of samples, via the Chao1, Shannon, Simpson, and the abundance-based coverage estimator indices. As shown in Table 4, the Good’s coverage for the two diets was 99.78%, indicating that the detection results for each sample were saturated. The BLE supplementation at 1.3 g/kg DM was associated with higher Shannon index (p = 0.07), and higher Chao 1 index (p = 0.06) than the control diet. However, no significant effect of BLE supplementation was found on the number of clean tags or Good’s coverage.

### β-Diversity

**Figure 2:**
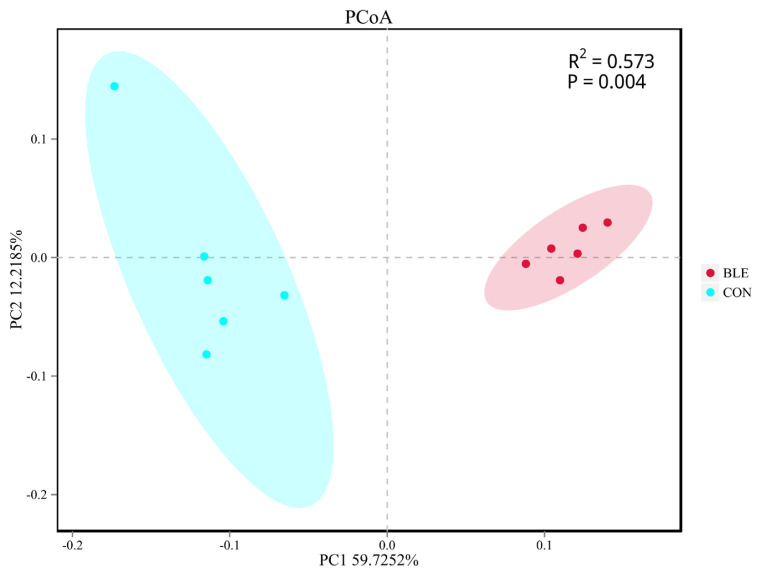
Principal components analysis (PCoA) of the rumen bacterial community. CON, total mixed ration supplemented with bamboo leaf flavonoid (BLE) at 0 g/kg dry matter; BLE, total mixed ration supplemented with BLE at 1.3 g/kg dry matter. The PCoA was based on the unweighted UniFrac distances between the microbiome profiles.

Beta diversity analysis was then conducted to evaluate the differences between the groups with regard to population complexity. PCoA, based on unweighted UniFrac distance metrics, was conducted to compare the bacterial profiles associated with the two treatments. As shown in Figure 2, PCoA axes 1 and 2 accounted for 59.72% and 12.22% of the total variation, respectively. Analysis of similarities (ANOSIM) yielded an R of 0.573 (p<0.01), which indicated that significant differences were found between each treatment group.

BLE supplementation affected the composition of the bacterial community as a whole, and therefore an analysis of the rumen bacteria was conducted at a number of levels to determine the effects of BLE on the abundances of rumen bacterial taxa. The results are shown in Table 5 and 6, at the levels of phylum and genus, respectively. Table 5 shows that *Firmicutes*, *Bacteroidetes*, and *Proteobacteria* were the three most abundant phyla and BLE supplementation at 1.3 g/kg DM was associated with larger *Proteobacteria* (p<0.01) and smaller *Firmicutes* (p<0.01) populations than the control diet. However, no significant effect of BLE supplementation was found on the size of the *Bacteroidetes* population. BLE also increased the size of the *Patescibacteria* (p<0.01), *Kiritimatiellaeota* (p = 0.08), and *Cyanobacteria* (p = 0.07) populations versus the control diet. At the genus level, and as shown in Table 6, *Prevotella_1*, *Weissella*, and *Succiniclasticum* were most abundant. BLE supplementation at 1.3 g/kg DM was associated with larger populations of *Butyrivibrio_2* (p<0.01), *Ruminococcus_2* (p<0.01), *Succiniclasticum* (p = 0.02), *Acinetobacter* (p<0.01), *Candidatus_Saccharimonas* (p<0.01), *Succinivibrionaceae_UCG-002* (p = 0.10), and *Clostridium_sensu_stricto_1* (p = 0.10); and a smaller population of *Weissella* (p<0.01) than the control diet.

### Relationships of bacterial communities at the phylum level with host production performance and ruminal fermentation parameters

**Figure 3:**
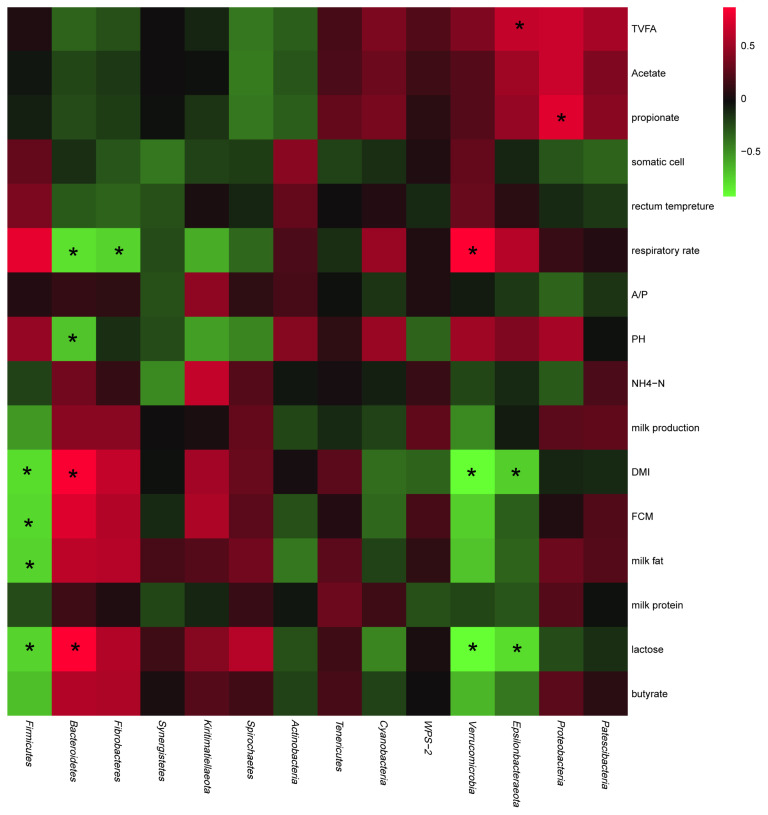
Relationships of the abundances of rumen bacteria at the phylum level with production performance and rumen fermentation parameters. Red represents a positive correlation and green represents a negative correlation. * p<0.05.

As shown in Figure 3, in terms of production performance, the abundances of *Epsilonbacteraeota*, *Verrucomicrobia*, and *Firmicutes* negatively correlated with DMI and milk yield, whereas those of *Bacteroidetes*, *Fibrobacteres*, *Kiritimatiellaeota*, and *Spirochaetes* positively correlated with these parameters. In terms of ruminal fermentation parameters, the abundances of *Spirochaetes* and *Actinobacteria* were negatively correlated with the TVFA, acetate, and propionate concentrations, whereas those of *Patescibacteria*, *Epsilonbacteraeota*, and *Proteobacteria* positively correlated with these parameters.

## DISCUSSION

### Production performance

Many studies of flavonoids have shown that alfalfa flavonoids increase milk yield and improve dairy quality, without affecting DMI [16]. Additionally, the feeding of a grape extract that is rich in flavonoids or a green tea and turmeric extract improves milk yield, milk fat, and milk protein content, without affecting feed intake [17]. In this study, BLE increased milk yield and milk fat content of heat-stressed cows, but had no effect on DMI. The present findings are, in general, consistent with those of previous studies. Thus, it is reasonable to suggest that bamboo leaf flavonoids as the major bioactive substance of BLE may have played a major role on improving production performance of heat-stressed cows.

During periods of heat stress, dairy cows reduce their heat generation by reducing their DMI, but this reduction in DMI creates a negative energy balance and reduces milk yield [18]. Recent research has shown that an approximate 30% reduction in DMI causes a 27.6% loss in milk yield, and 50% of this milk loss can be attributed to changes in the rumen bacterial communities and metabolic disorders [19]. In this study, BLE significantly reduced the abundance of *Firmicutes* and increased *Bacteroidetes* and *Proteobacteria*. Spearman correlations analysis showed that *Firmicutes* and production performance were negatively correlated. However, *Bacteroidetes* and production performance were positively correlated. Xu and Guo [20] reported that milk fat content of dairy cows decreased significantly after starch supplementation, *Firmicutes* increased significantly and *Bacteroidetes* showed a trend of reduction. Studies showed that rumen acetate concentration is an important index to determine the milk fat content. In this study, *Firmicutes* and rumen acetate concentration were negatively correlated but there was no significant difference. It was worth noting that in this study, *Proteobacteria* has a significant positive correlation with the concentration of acetate. BLE significantly increased the abundance of *Proteobacteria*, improved rumen acetate concentration, and thus improved the production performance of cows.

Studies showed that *Staphylococcus aureus* is the most frequent cause of cow mastitis and accounts for more than 50% of the cases [21]. The high temperature environment in summer was conducive to the growth of *Staphylococcus aureus*, which enters through the mammary duct of cows and sticks to the mammary gland tissue, thus inducing mastitis in cows. Dairy cows with mastitis produced a high SCC that entered the milk. Therefore, SCC was an important indicator for evaluating cow mastitis. In the present study, the SCC of the BLE-supplemented cows was lower than control group. Studies showed that BLE may play a role in intercalation or hydrogen bonding with the stacking of nucleic acid bases and produce an inhibitory action on DNA and RNA synthesis, consequently inhibiting the growth of *Staphylococcus aureus* [22]. This indicated that the addition of BLE to the diet could reduce the risk of mastitis during periods of heat stress.

### Rumen fermentation parameters

In the present study, the supplementation of BLE increased the rumen TVFA, acetate, butyrate, and valerate concentrations. Rumen TVFA concentration can serve as an indicator of energy balance and energy utilization in cows [7]. A high rumen TVFA concentration improves the milk yield by cows, and in the present study, the increase in TVFA associated with BLE was also associated with an increase in milk yield. Acetate is the predominant substrate for *de novo* fatty acid synthesis in dairy cows because it serves as a 2-carbon donor for the synthesis of malonyl-CoA and for nicotinamide adenine dinucleotide phosphate synthesis through the isocitrate pathway [23]. Additionally, in well-fed cows, acetate is the major VFA produced in the rumen and provides 45% of the energy arising from VFA metabolism [7]. Acetate is absorbed into the blood through the rumen wall and is used as a substrate for the de novo synthesis of fatty acids by mammary epithelial cells, which are included in the milk fat [24]. Therefore, acetate is required for dairy cows to meet their energy requirements and for milk fat synthesis. However, of all the milk components, milk fat is the most variable and is highly influenced by dietary nutrient composition [25]. BLE supplementation at 1.3 g/kg DM was associated with higher rumen acetate and butyrate concentrations than consumption of the control diet. This likely explains the increase in milk fat content and 4% FCM in the present study. Additionally, we speculated that dietary BLE would promote the growth of favorable rumen bacteria under conditions of environmental stress, which would result in an increase in the rumen TVFA, acetate, and butyrate concentrations.

### Rumen bacterial communities

Zhan et al [16] reported that alfalfa flavonoids have a protective effect and promote the growth of favorable bacteria in the rumen under conditions of environmental stress. The results of the present study are consistent with these previous findings. In the present study, BLE significantly increased the Chao 1 index, and Shannon index versus the control diet. This indicates that BLE significantly increases the rumen bacterial abundance and the diversity of the rumen bacterial community. Previous studies have shown that the *Firmicutes* and *Bacteroidetes* are the most abundant phyla in the rumen of ruminants [9]. A low *Firmicutes*/*Bacteroidetes* ratio is known to be required for the efficient artificial degradation of lignocelluloses [26] and Do et al [27] found that a low *Firmicutes*/*Bacteroidetes* ratio in the rumen is associated with more efficient lignocellulose digestion. In the present study, BLE significantly reduced the *Firmicutes*/*Bacteroidetes* ratio in the rumen. On the basis of these findings, we speculate that BLE may maintain a low *Firmicutes*/*Bacteroidetes* ratio in the rumens of heat-stressed dairy cows, resulting in improved fermentation of fiber and higher rumen TVFA, acetate, butyrate, and valerate concentrations. At the genus level, *Bacteroides*, *Butyrivibrio*, *Ruminococcu*s, and *Fibrobacter* were the dominant fibrolytic microorganisms. The enzymes produced by these microbes have been reported to digest plant polymers, such as cellulose, hemicellulose, and oligosaccharide [7]. In the present study, BLE supplementation at 1.3 g/kg DM was associated with larger populations of *Butyrivibrio_2* and *Ruminococcus_2*. Therefore, these findings suggest that BLE may also promote the growth of bacterial genera that ferment fiber.

### Conclusions

Supplementation with 1.3 g BLE/kg DM during a period of heat stress increases milk yield, milk fat content, and the rumen concentrations of TVFA, acetate, butyrate, and valerate. Furthermore, this superior rumen fermentation can be at least partially attributed to more appropriate proportions of *Firmicutes*, *Bacteroidetes*, *Butyrivibrio_2*, and *Ruminococcus_2*. Therefore, BLE is a natural plant extract that may improve production performance and rumen fermentation during periods of heat stress and provide new strategies for relieving the deleterious effects of heat stress in dairy cows.

## Figures and Tables

**Table 1:** Ingredients and chemical composition of the total mixed ration (dry matter basis)

Items	Content
Ingredients (%)	
Corn silage	24.5
Corn	15.7
Cottonseed meal	3.3
Alfalfa hay	14.3
Leymus chinensis	10.2
Distillers dried grains with solubles	3.1
Steam-flaked corn	8.2
Soybean meal	12.3
Beet pulp	4.8
Premix^1)^	3.0
NaCl	0.6
Total	100
Chemical composition (%)	
NEL (MJ/kg)^2)^	7.13
EE	4.56
CP	17.36
ADF	18.52
NDF	31.34
Ca	0.68
P	0.41

NE_L_, net energy for lactation; EE, ether extract; CP, crude protein; ADF, acid detergent fiber; NDF, neutral detergent fiber; Ca, calcium; P, phosphorus.

1) One kilogram of the premix contained the following: Fe, 1,400 mg; Cu, 1,200 mg; Mn, 2,400 mg; Zn, 5,500 mg; Se, 40 mg; Co, 30 mg; I, 90 mg, vitamin A, 900,000 IU; vitamin D, 700,000 IU; vitamin E, 9,000 IU.

2) NE_L_, calculated according to NRC (2001).

**Table 2:** Effects of dietary bamboo leaf flavonoid supplementation on dry matter intake and milk yield and composition during heat stress in Holstein dairy cows

Items	CON^1)^	BLE^1)^	SEM	p-value
Dry matter intake (kg/d)	20.63	20.58	0.23	0.80
Yield (kg/d)				
Milk	36.03	37.11	0.49	<0.01
Fat	1.18	1.38	0.02	0.04
Protein	1.16	1.18	0.04	0.46
Lactose	1.86	1.90	0.06	0.53
4% Fat-corrected milk^2)^	32.11	35.54	0.53	<0.01
Milk content (%, unless otherwise stated)				
Fat	3.27	3.72	0.07	<0.01
Protein	3.23	3.19	0.13	0.18
Lactose	5.16	5.13	0.08	0.37
Somatic cells count (×10^4^/mL)	18.54	12.33	1.25	<0.01

n = 6 per group. The same as below.

BLE, bamboo leaf flavonoid; SEM, standard error of the mean.

1) CON, total mixed ration supplemented with BLE at 0 g/kg dry matter; BLE, total mixed ration supplemented with BLE at 1.3 g/kg dry matter.

2) 4% fat-corrected milk = 0.4×milk (kg/d)+15×fat (kg/d).

**Table 3:** Effects of bamboo leaf flavonoid supplementation on rumen fermentation parameters during heat stress in Holstein dairy cows

Items	CON^1)^	BLE^1)^	SEM	p-value
Rumen pH	6.68	6.65	0.04	0.66
Ammonia nitrogen (mg/dL)	15.03	15.07	0.35	0.45
Rumen volatile fatty acids (mmol/L)				
Total volatile fatty acids	94.52	109.87	1.86	<0.01
Acetate	56.75	65.82	0.97	<0.01
Propionate	23.40	26.64	0.54	0.21
Acetate/propionate	2.88	2.85	0.08	0.64
Butyrate	10.38	13.20	0.23	<0.01
Isobutyrate	0.95	0.95	0.03	0.93
Valerate	1.28	1.50	0.11	0.05
Isovalerate	1.76	1.77	0.06	0.81

BLE, bamboo leaf flavonoid; SEM, standard error of the mean.

1) CON, total mixed ration supplemented with BLE at 0 g/kg dry matter; BLE, total mixed ration supplemented with BLE at 1.3 g/kg dry matter.

**Table 4:** Number of clean tags, operational taxonomic units, and alpha diversity indexes of the rumen bacteria

Items	CON^1)^	BLE^1)^	SEM	p-value
Clean tags	56,970	60,784	58.36	0.54
Good’s coverage %	99.78	99.78	0.06	0.34
Shannon index	4.20	4.71	0.33	0.07
Chao 1 index	853.46	891.55	15.26	0.06

BLE, bamboo leaf flavonoid; SEM, standard error of the mean.

1) CON, total mixed ration supplemented with BLE at 0 g/kg dry matter; BLE, total mixed ration supplemented with BLE at 1.3 g/kg dry matter.

**Table 5:** Effects of bamboo leaf flavonoid supplementation on the relative abundance of rumen bacteria at the phylum level during heat stress in Holstein dairy cows

Items	CON^1)^	BLE^1)^	SEM	p-value
*Firmicutes*	6,980.51	5,649.11	0.32	<0.01
*Bacteroidetes*	2,503.29	2,782.43	0.46	0.17
*Proteobacteria*	172.43	1,095.63	0.26	<0.01
*Patescibacteria*	183.10	298.14	0.25	<0.01
*Kiritimatiellaeota*	17.34	19.26	0.55	0.08
*Spirochaetes*	32.08	31.41	0.86	0.76
*Actinobacteria*	47.92	47.46	0.43	0.91
*Tenericutes*	21.06	21.70	0.38	0.74
*Cyanobacteria*	18.33	19.96	0.59	0.07
*WPS-2*	6.60	6.33	0.88	0.70
*Synergistetes*	17.77	17.51	0.87	0.87
*Fibrobacteres*	0.78	0.79	0.95	0.88
*Epsilonbacteraeota*	4.53×10^−4^	4.79×10^−4^	0.26	0.34
*Verrucomicrobia*	7.13×10^−4^	8.00×10^−4^	0.15	0.47

BLE, bamboo leaf flavonoid; SEM, standard error of the mean.

1) CON, total mixed ration supplemented with BLE at 0 g/kg dry matter; BLE, total mixed ration supplemented with BLE at 1.3 g/kg dry matter.

**Table 6:** Effects of bamboo leaf flavonoid supplementation on the relative abundance of rumen bacteria at the genus level during heat stress in Holstein dairy cows

Items	CON^1)^	BLE^1)^	SEM	p-value
*Prevotella_1*	1,367.6	1,442.02	0.47	0.63
*Weissella*	3,126.19	1,279.89	0.53	<0.01
*Rikenellaceae_RC9_gut_group*	453.97	451.02	0.16	0.81
*Succiniclasticum*	760.55	931.33	0.25	0.02
*uncultured_bacterium_f_Muribaculaceae*	202.25	168.05	0.22	0.11
*uncultured_bacterium_f_F082*	316.44	360.22	0.35	0.11
*Ruminococcaceae_NK4A214_group*	386.73	392.62	0.68	0.91
*Streptococcus*	587.09	593.99	0.69	0.84
*Butyrivibrio_2*	189.39	204.17	0.88	<0.01
*Christensenellaceae_R-7_group*	294.51	255.70	0.48	0.36
*Lactobacillus*	525.68	176.36	0.57	<0.01
*Acinetobacter*	37.13	441.03	0.16	<0.01
*Candidatus_Saccharimonas*	177.69	292.51	0.35	<0.01
*Succinivibrionaceae_UCG-002*	0.58	0.75	0.52	0.10
*Ruminococcaceae_UCG-014*	128.57	132.26	0.44	0.80
*Selenomonas_1*	8.33	7.63	0.63	0.33
*Ruminococcus_2*	58.08	70.02	0.29	<0.01
*Clostridium_sensu_stricto_1*	135.95	142.73	0.54	0.10
*Lachnospiraceae_NK3A20_group*	73.77	71.66	0.16	0.41
Others	1,621.33	1,882.86	0.59	0.11

BLE, bamboo leaf flavonoid; SEM, standard error of the mean.

1) CON, total mixed ration supplemented with BLE at 0 g/kg dry matter; BLE, total mixed ration supplemented with BLE at 1.3 g/kg dry matter.
